# WHO guidance for asthma and chronic obstructive pulmonary disease: why it matters

**DOI:** 10.2471/BLT.26.296819

**Published:** 2026-08-01

**Authors:** Tara Ballav Adhikari, Mohammad-Mahdi Rashidi, Patricia Alupo, Alarcos Cieza, Sarah Rylance

**Affiliations:** a Department of Noncommunicable Diseases and Mental Health, World Health Organization, Avenue Appia 20, 1211 Geneva 27, Switzerland.

Asthma and chronic obstructive pulmonary disease are major global health conditions, affecting over 500 million people and causing around 4 million deaths each year.^1^ Together, they account for approximately 100 million disability-adjusted life years, with important social and economic consequences, including lost productivity, disrupted education and financial strain for affected households.^1^ The burden falls disproportionately on low- and middle-income countries, reflecting persistent inequities in access to diagnosis and care.^2^

Addressing these gaps requires strengthening primary health care, yet major constraints remain, particularly in access to diagnostic tools, essential inhaled medicines and structured follow-up. Many patients cannot access evidence-based care at first contact, and chronic respiratory diseases remain underdiagnosed and poorly controlled, leading to avoidable morbidity and mortality.^2^

Progress towards global targets has been limited. Although the World Health Organization (WHO) *Global action plan for the prevention and control of noncommunicable diseases 2013–2020*^3^ set a national target of at least 80% availability of affordable essential medicines and basic technologies required to treat major noncommunicable diseases, substantial gaps persist. Bronchodilator inhalers are available in public-sector primary care in 97% (57/59) of high-income countries compared to 48% (12/25) of low-income countries.^4^ Spirometry is available in only 40% (77/193) of countries overall and in just 4% (1/25) of low-income settings.^4^ Inhaled medicines, especially corticosteroids, are often unavailable or unaffordable in resource-constrained settings, leading to continued reliance on outdated oral therapies and highlighting broader system inequities.^5^

To accelerate progress towards universal health coverage (UHC), countries need practical guidance to integrate chronic respiratory diseases into primary care systems.^6^^,^^7^ While the *WHO package of essential noncommunicable disease interventions for primary health care*^8^ provides a foundation, its chronic respiratory disease protocols have remained unchanged since the original 2010 edition. At the same time, the *WHO Model list of essential medicines* has included newer inhaled therapies such as the budesonide-formoterol combination inhaler since 2017, creating a disconnect between policy tools. Updated WHO guidance is therefore needed to provide coherent, evidence-based recommendations aligned with current standards of care. WHO is currently developing such guidance for asthma and chronic obstructive pulmonary disease in primary care, expected to be published in early 2027.

WHO guideline development is designed to be transparent, methodologically rigorous, free from conflict of interest and responsive to diverse health system contexts. Recommendations are informed by systematic evidence reviews, assessments and frameworks that consider feasibility, acceptability, equity and resource implications alongside clinical effectiveness.^9^

A key strength of this process is the composition of guideline development groups, which reflect geographic, professional and income-level diversity.^9^ Members include primary care providers, nurses, pharmacists, physiotherapists, specialists, policy-makers and people with lived experience, drawn from all WHO regions. This mix ensures recommendations are globally relevant and grounded in real-world constraints. Priority questions are shaped through consultation across WHO and guideline development group members to reflect policy needs.

Non-WHO guidelines for chronic respiratory diseases play an important role in synthesizing clinical evidence and defining standards of care. However, such guidelines are frequently based on high-income contexts and may assume availability of advanced diagnostics and infrastructure. While scientifically robust, these assumptions can limit their applicability in lower-resource settings.^10^ Forthcoming WHO guidance for chronic respiratory diseases will complement these efforts and bridge the gap between clinical evidence and implementation, offering a policy-oriented perspective on equity, feasibility and integration into primary care.

Importantly, the forthcoming guidelines will link chronic respiratory care with global commitments to UHC and sustainable development goal target 3.4. The 2025 United Nations political declaration calls for scaling up primary health-care services, aiming for 80% availability of essential medicines and technologies by 2030.^6^ Similarly, World Health Assembly resolution WHA.78.5^7^ urges countries to integrate lung health within primary health-care systems, consistent with the equity and resilience priorities of the WHO Fourteenth General Programme of Work.^11^

The guidelines will help reposition asthma and chronic obstructive pulmonary disease as public health priorities that can be routinely managed within primary care rather than specialist settings. At country level, updated guidance can be locally adapted to support decisions on diagnosis, treatment, referral and follow-up within routine primary care services. The updated guidelines can inform national essential medicines lists, benefit packages, procurement strategies and clinical protocols, and will be particularly valuable in settings where access to diagnostics and inhaled therapies remains limited.

However, guideline publication alone will not improve outcomes. Impact will depend on effective adaptation, integration into health systems and sustained investment in primary health care.^12^ The success of the guidelines will be measured by how effectively they enable countries to translate evidence into equitable, implementable care at scale.
